# The efficacy of enteral nutrition combined with accelerated rehabilitation in non-small cell lung cancer surgery: A randomized controlled trial protocol

**DOI:** 10.1097/MD.0000000000023382

**Published:** 2020-11-25

**Authors:** Xiaona Ji, Haiyan Ding

**Affiliations:** a Department of Thoracic Surgery, Cancer Hospital Affiliated to Shandong First Medical University; b Department of Intensive-Care Unit, Jinan Children's Hospital, Shandong, China.

**Keywords:** accelerated rehabilitation, enteral nutrition, non-small cell lung cancer, protocol

## Abstract

**Objective:**

To investigate the effect of enteral nutrition combined with accelerated rehabilitation in treating the non-small cell lung cancer (NSCLC).

**Methods:**

It is a randomized controlled experiment to be carried out from June 2021 to December 2021. It was permitted through the Ethics Committee of Cancer Hospital Affiliated to Shandong First Medical University (00923876). 100 patients are included in the study. The inclusion criteria contain: (1) patients with NSCLCs receiving surgery as the primary treatment; (2) over 18 years of age. The exclusion criteria are as follows: (1) age ≥65 years; (2) severe metabolic and systemic diseases, such as diabetes, hypertension, or severe liver and kidney dysfunction; (3) the body mass index <18.5 kg/m^2^; (4) patients who have received preoperational radiotherapy or chemotherapy. Patients in the control group are provided routine nutrition, including preoperative nutritional risk screening and assessment and preoperative nutrition education and dietary guidance, while patients in the nutrition group are provided additional enteral nutrition preparations combined with accelerated rehabilitation as in the control group. The primary outcomes include the perioperative change of serum albumin, serum prealbumin, hemoglobin, and total lymphocyte counts. The second outcomes include length of hospitalization, quality of life, and risk of postoperative complications.

**Results:**

Table 1 shows the comparison of indicators after surgery between the 2 groups.

**Conclusion:**

Enteral nutrition combined with accelerated rehabilitation appears to be beneficial in decreasing the complications and improving postoperative recovery after NSCLC surgery.

## 1 Introduction

Lung cancer is the main cause of the mortality related to cancer worldwide.^[1,2]^ Approximately 85% of lung cancer patients are confirmed as the non-small cell lung cancer (NSCLC).^[3]^ Nearly one out of every four cancer-related deaths is caused by NSCLC, which is more than the sum of colon cancer, prostate cancer, and breast cancer.^[4]^ NSCLC can be divided into large cell carcinoma, squamous cell carcinoma, and the adenocarcinoma.^[5]^

The American Cancer Society has estimated that about 222,000 new lung cancer cases will be diagnosed in 2017, and more than 155,000 patients will die from lung cancer. At the time of treatment, most patients are diagnosed with the advanced disease. Over the last decade, significant advances in the molecular characterization of lung cancer have resulted in considerable progress in treatment planning and have led to the creation of effective targeted therapies.^[6–8]^ At present, the main treatment for NSCLC is surgery, and with the development of minimally invasive endoscopic treatment technology, thoracoscopic lobectomy is increasingly being used in the surgical treatment of early lung cancer and pulmonary nodules.^[9,10]^ The patients’ nutritional condition offers the basis for postoperative recovery. From the first evaluation of patient to the early postoperative period, there are opportunities to optimize the results. Enteral nutrition and accelerated rehabilitation is a new perioperative multidisciplinary treatment developed on the basis of medical evidence that not only reduces the stress and trauma experienced by the patient but also ensures the nutritional supply needed to maintain the state of hypermetabolism during trauma, thus achieving the goal of rapid recovery. However, few studies have reported relevant topics. Thus, we perform this a randomized controlled trial protocol to investigate the effect of enteral nutrition combined with accelerated rehabilitation in treating the NSCLC to provide a basis for better clinical nutrition in the future.

## 2 Methods

### 2.1 Study design

It is a randomized controlled experiment to be conducted from June 2021 to December 2021. It was permitted through the Ethics Committee of Cancer Hospital Affiliated to Shandong First Medical University (00923876), and this experiment was registered with research registry (researchregistry6157). Patients are assigned randomly to nutrition group and control group. When patients arrive in operating room, they are randomly divided into groups using sealed, opaque, and numbered envelopes. These envelopes are generated by applying the computer-generated randomized list. The relevant nurses, surgeons, and all the patients are not aware of it.

### 2.2 Inclusion and exclusion criteria

This study includes a total of 100 patients. The inclusion criteria contain:

(1) patients with NSCLCs receiving surgery as the primary treatment;
(2) over 18 years of age.

The exclusion criteria are as follows:

(1) age ≥65 years;
(2) severe metabolic and systemic diseases, such as diabetes, hypertension, or severe liver and kidney dysfunction;
(3) the body mass index <18.5 kg/m^2^;
(4) patients who have received preoperational radiotherapy or chemotherapy.

### 2.3 Intervention

Patients in the control group are provided routine nutrition, including preoperative nutritional risk screening and assessment and preoperative nutrition education and dietary guidance, while patients in the nutrition group are provided additional enteral nutrition preparations combined with accelerated rehabilitation as in the control group. The main methods are

(1) health education. After hospitalization, patients are given health education for diseases according to their educational level, and a risk assessment is made according to their physical condition, medical history, and nutritional status to ensure that patients fully understood their own physical condition, thereby improving the success rate of surgery.
(2) Preoperative preparation. The control group is deprived of water 8 hours before surgery, while the nutrition group receives 10% glucose solution (1000 mL) 1 night before operation and then 200 mL of 10% glucose solution 2 hours before surgery.
(3) Postoperative nutritional intervention. The control group sits up one day after the operation and are fed a semi-liquid diet, eventually transitioning to a normal diet 2 days after the operation. The patients are encouraged to eat and given dietary guidance.

In the nutrition group, sitting up is encouraged, and 200 mL of 5% glucose solution is given in the evening on the same day of the operation. One day after the operation, patients are instructed to eat normally and increase bed activity to assist with sputum expectoration. Three days after the operation, 400 mL of enteral nutrition is given orally based on a normal diet.

### 2.4 Outcomes

The primary outcomes include the perioperative change of serum albumin, serum prealbumin, hemoglobin, and total lymphocyte counts. The second outcomes include length of hospitalization, quality of life, and risk of postoperative complications.

### 2.5 Statistical analysis

The analysis of data is carried out with the software of IBM SPSS Statistics for Windows, version 20 (IBM Corp., Armonk, NY, USA). Afterward, all the data are described with appropriate characteristics such as mean, median, standard deviation as well as percentage. Continuous and categorical variables are analyzed using χ^2^-tests and independent *t*-tests, respectively. *P* value of less than .05 indicates that there is statistical significance.

## 3 Results

Table 1 shows the comparison of indicators after surgery between the 2 groups.

**Table 1:** Comparison of indicators after surgery between the 2 groups.

Variables	Nutrition group (n = 50)	Control group (n = 50)	*P* value
Serum albumin (g/L)			
Serum prealbumin (g/L)			
Hemoglobin (g/L)			
Total lymphocyte counts (10^9^/L)			
Length of hospitalization (d)			
Quality of life			
Postoperative complications (n)			

## 4 Discussion

In thoracic surgery, the nutritional risk of patients after thoracotomy is mainly related to surgical stress.^[11]^ Changes in the body's internal environment induced by surgical stress can lead to glucose, protein, and fat metabolism disorders.^[12,13]^ In the cancer treatment and surgery, the timing, form, and degree of the nutritional support are significant, which is a serious catabolic burden process. This is especially important considering that malnutrition is a very significant factor affecting the postoperative incidence rate and mortality, which can be seen in 20 to 50 percent of patients.^[14,15]^ The core of the concept of enteral nutrition combined with accelerated rehabilitation surgery is to reduce the surgical stress response by strengthening psychological counseling before and after surgery, improving anesthesia, administering unconventional bowel preparation, paying attention to intraoperative heat preservation, reducing water and sodium retention, implementing early extubation and promoting early out-of-bed activities. Mechanical bowel preparation is not only a stressor but also results in electrolyte imbalance and dehydration, particularly in the elderly patients. Studies have found that bowel preparation is not beneficial to the patients receiving colon surgery, and it may enhance the risk of anastomotic leakage after surgery.^[16]^ Therefore, in the concept of accelerated rehabilitation surgery, preoperative fasting is no longer required, and patients are encouraged to consume oral sugar-containing liquids before surgery. The application of nutritional management is simple and should be audited to improve the adherence and effectiveness of the programs to promote the rehabilitation and reduce perioperative complications.

## 5 Conclusion

Enteral nutrition combined with accelerated rehabilitation appears to be beneficial in decreasing the complications and improving postoperative recovery after NSCLC surgery.

## Author contributions

**Funding acquisition:** Xiaona Ji.

**Investigation:** Haiyan Ding.

**Methodology:** Haiyan Ding.

**Software:** Haiyan Ding.

**Writing – original draft:** Xiaona Ji.

**Writing – review & editing:** Xiaona Ji.
